# Supplementary material for: Triglyceride and Glucose Index Predicts Acute Coronary Syndrome in Patients with Antineutrophil Cytoplasmic Antibody-Associated Vasculitis
Source: Diagnostics (Basel). 2022 Jun 17;12(6):1486. doi: 10.3390/diagnostics12061486 (PMC9221824; doi:10.3390/diagnostics12061486)
Supplement: Supplementary file 1 [file diagnostics-12-01486-s001.zip › diagnostics-1695880-supplementary.pdf]

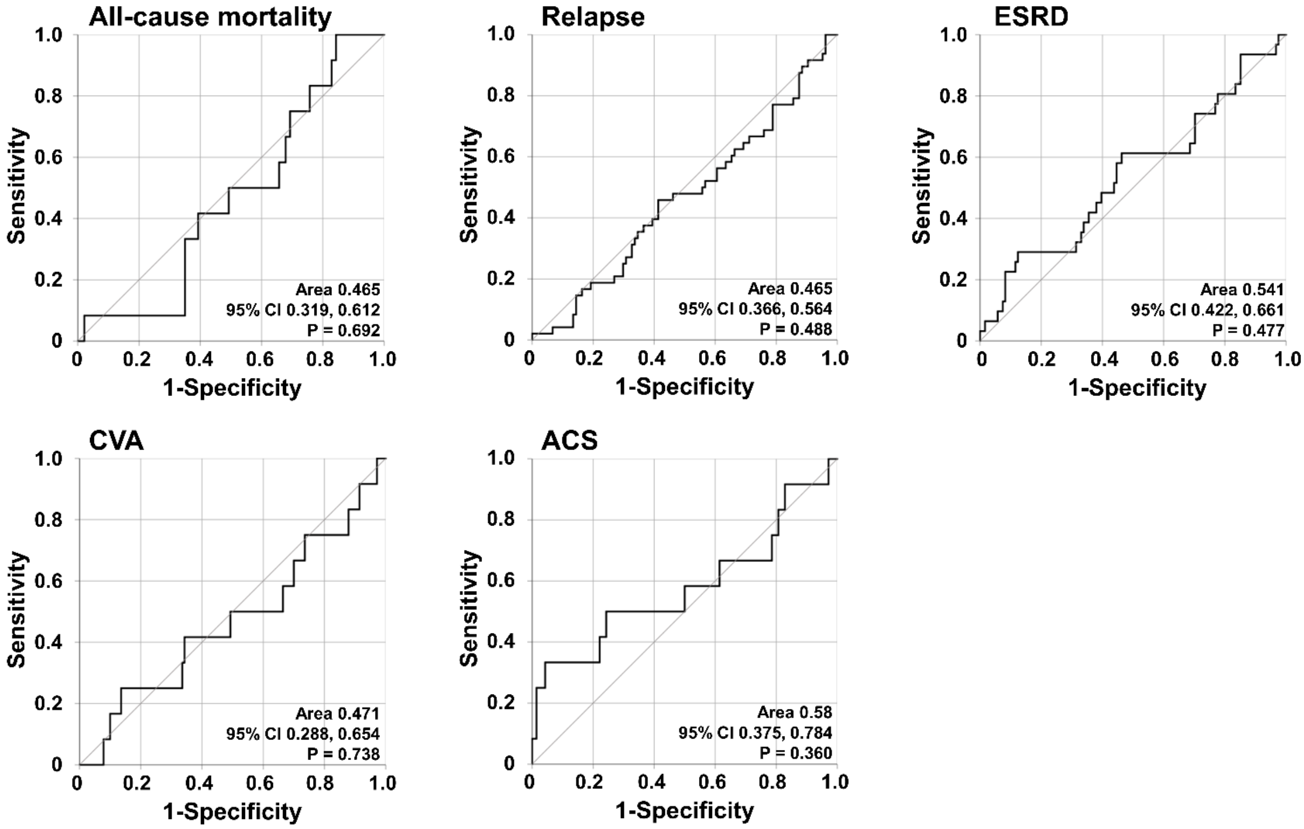

Supplementary Figure S1. The ROC curve analysis of TyG index for all-cause mortality, relapse, ESRD, CVA and ACS in patients with AAV. ROC, receiver operator characteristic; TyG, triglyceride-glucose; ESRD, end-stage renal disease; CVA, cerebrovascular accident; ACS, acute coronary syndrome; AAV, ANCA-associated vasculitis; ANCA, antineutrophil cytoplasmic antibody.

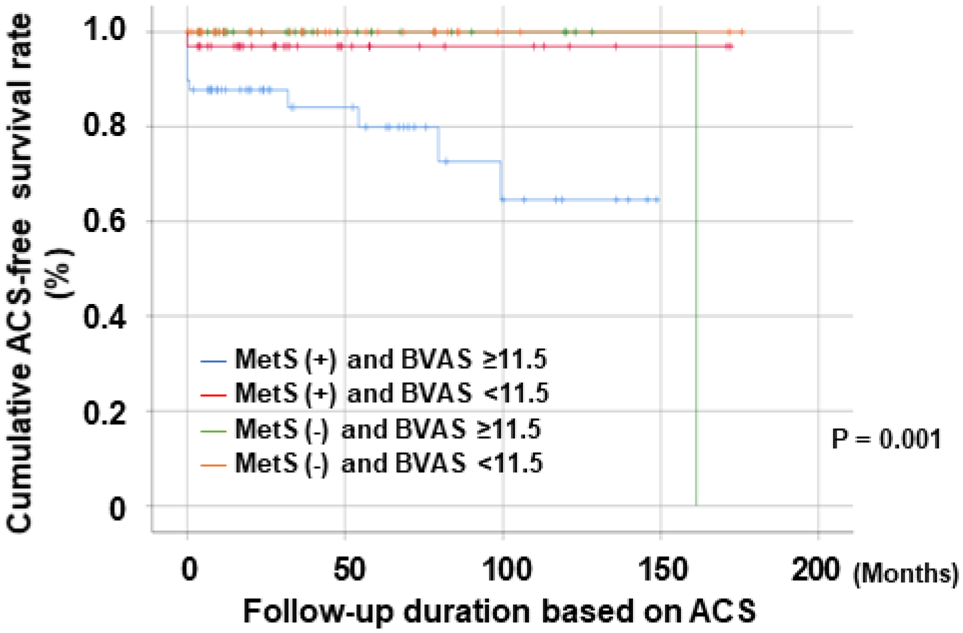

Supplementary Figure S2. Comparison of the cumulative ACS-free survival rates among the four groups according to the presence of MetS and BVAS ≥11.5. ACS, acute coronary syndrome; MetS, metabolic syndrome; BVAS, Birmingham vasculitis activity score.

Supplementary Table S1. Correlation of the TyG index with continuous variables at AAV diagnosis

**Variables**	**Values**	
	**Correlation coef ficient**	**P-value**
Age (years)	0.118	0.146
BMI (kg/m^2)	0.222	0.006
BVAS	0.019	0.819
FFS	0.063	0.447
White blood cell count (/mm^3)	0.076	0.354
Haemoglobin (g/dL)	-0.006	0.941
Platelet count (× 1000/mm^3)	0.083	0.314
Blood urea nitrogen (mg/dL)	0.026	0.750
Serum creatinine (mg/dL)	0.077	0.347
Serum albumin (g/dL)	-0.063	0.448
Total cholesterol (mg/dL)	0.002	0.976
HDL-cholesterol (mg/dL)	-0.182	0.029
LDL-cholesterol (mg/dL)	-0.236	0.005
ESR (mm/hr)	0.134	0.104
CRP (mg/L)	0.081	0.325

TyG, triglyceride glucose; AAV, ANCA-associated vasculitis; ANCA, antineutrophil cytoplasmic antibody; BMI, body mass index; BVAS, Birmingham vasculitis activity score; FFS, five factor score; HDL, high density lipoprotein; LDL, low density lipoprotein; ESR, erythrocyte sedimentation rate; CRP, C-reactive protein.

Supplementary Table S2. Logistic regression analysis of variables for metabolic syndrome in AAV patients at the time of diagnosis*

**Variables**	**Univariable**			**Multivariable (Variables with P < 0.10)**		
	**OR**	**95% CI**	**P value**	**OR**	**95% CI**	**P value**
Age	1.038	1.014, 1.064	0.002	1.026	0.989, 1.064	0.168
Male sex	1.131	0.573, 2.234	0.722			
BMI	1.268	1.123, 1.432	<0.001	1.327	1.131, 1.556	0.001
MPA	0.967	0.509, 1.836	0.918			
GPA	1.018	0.477, 2.172	0.963			
EGPA	1.031	0.475, 2.237	0.938			
MPO-ANCA (or P-ANCA) positive	2.636	1.313, 5.291	0.006	1.592	0.603, 4.204	0.348
PR3-ANCA (or C-ANCA) positive	0.671	0.271, 1.664	0.389			
BVAS	1.060	1.010, 1.113	0.019	1.046	0.970, 1.127	0.240
FFS	1.363	0.997, 1.864	0.053	0.778	0.454, 1.333	0.360
White blood cell count	1.000	1.000, 1.000	0.050	1.000	1.000, 1.000	0.396
Haemoglobin	0.822	0.711, 0.950	0.008	0.723	0.538, 0.971	0.031
Platelet count	1.003	1.001, 1.006	0.013	1.003	0.999, 1.006	0.145
Blood urea nitrogen	1.011	0.996, 1.026	0.140			
Serum creatinine	1.093	0.928, 1.287	0.287			
Serum albumin (g/dL)	0.529	0.326, 0.857	0.010	1.710	0.616, 4.747	0.303
Total cholesterol (mg/dL)	0.998	0.991, 1.005	0.531			
ESR (mm/hr)	1.014	1.004, 1.023	0.004	0.997	0.982, 1.012	0.700
CRP (mg/L)	1.010	1.003, 1.018	0.004	1.005	0.994, 1.015	0.396
**TyG**	6.572	2.943, 14.677	<0.001	5.667	2.071, 15.506	0.001

*Since T2DM, hypertension, HDL-cholesterol, and LDL-cholesterol are included in the 5 components for the diagnosis of MetS, they were not included in the logistic regression analysis of variables for metabolic syndrome in AAV patients at diagnosis.

AAV, ANCA-associated vasculitis; ANCA, antineutrophil cytoplasmic antibody; BMI, body mass index; MPA, microscopic polyangiitis; GPA, granulomatosis with polyangiitis; EGPA, eosinophilic granulomatosis with polyangiitis; MPO, myeloperoxidase; P, perinuclear; PR3, proteinase 3; C, cytoplasmic; BVAS, Birmingham vasculitis activity score; FFS, five-factor score; ESR, erythrocyte sedimentation rate; CRP, C-reactive protein; TyG, triglyceride and glucose index.

Supplementary Table S3. Cox hazards model analysis of variables at AAV diagnosis for ACS during follow-up including BVAS ≥11.5

Variables	Univariable			Multivariable		
	**HR**	**95% CI**	**P value**	**HR**	**95% CI**	**P value**
Age	1.024	0.978, 1.071	0.309			
Male sex	4.933	1.403, 17.345	0.013	5.034	1.229, 20.619	0.025
BMI	1.131	0.940, 1.359	0.192			
MPA	1.595	0.475, 5.354	0.450			
GPA	0.730	0.159, 3.340	0.685			
EGPA	0.674	0.144, 3.151	0.616			
MPO-ANCA (or P-ANCA) positive	2.930	0.622, 13.804	0.174			
PR3-ANCA (or C-ANCA) positive	1.954	0.249, 15.314	0.524			
BVAS ≥11.5	11.112	1.376, 89.721	0.024	9.113	0.940, 88.349	0.057
FFS	1.936	1.147, 3.265	0.013	1.391	0.785, 2.468	0.258
T2DM	4.255	1.245, 14.537	0.021	1.707	0.464, 6.275	0.421
Hypertension	3.086	0.971, 9.805	0.056	2.789	0.809, 9.612	0.104
White blood cell count	1.000	1.000, 1.000	0.320			
Haemoglobin	0.807	0.615, 1.059	0.123			
Platelet count	1.000	0.997, 1.004	0.842			
Blood urea nitrogen	1.013	1.000, 1.027	0.054	1.005	0.989, 1.021	0.563
Serum creatinine	1.142	0.911, 1.432	0.249			
Serum albumin	0.600	0.273, 1.320	0.204			
Total cholesterol	1.005	0.992, 1.017	0.456			
HDL-cholesterol (mg/dL)	0.986	0.954, 1.019	0.401			
LDL-cholesterol (mg/dL)	1.007	0.993, 1.020	0.318			
ESR	1.007	0.992, 1.022	0.350			
CRP	1.005	0.997, 1.013	0.249			
TyG ≥ 9.011	3.054	0.959, 9.726	0.059	2.642	0.631, 11.064	0.184

AAV, ANCA-associated vasculitis; ANCA, antineutrophil cytoplasmic antibody; BMI, body mass index; MPA, microscopic polyangiitis; GPA, granulomatosis with polyangiitis; EGPA, eosinophilic granulomatosis with polyangiitis; MPO, myeloperoxidase; P, perinuclear; PR3, proteinase 3; C, cytoplasmic; BVAS, Birmingham vasculitis activity score; FFS, five-factor score; HDL, high density lipoprotein; LDL, low density lipoprotein; ESR, erythrocyte sedimentation rate; CRP, C-reactive protein; TyG, triglyceride and glucose.
